# Infrared Laser Effects on Cell Projection Depend on Irradiation Intermittence and Cell Activity

**DOI:** 10.3390/cells12040540

**Published:** 2023-02-08

**Authors:** Norma Medina-Villalobos, Remy Avila, María Marsal, Jordi Andilla, Pablo Loza-Álvarez, Mario Miguel Ojeda-Ramírez, Elisa Tamariz

**Affiliations:** 1 Instituto de Ciencias de la Salud, Universidad Veracruzana, Xalapa 91190, Veracruz, Mexico; 2 Centro de Física Aplicada y Tecnología Avanzada, Universidad Nacional Autónoma de México (UNAM), A.P. 1-1010, Juriquilla 76000, Querétaro, Mexico; 3 ICFO-Institut de Ciencies Fotoniques, The Barcelona Institute of Science and Technology, 08860 Castelldefels, Barcelona, Spain; 4 Facultad de Estadística e Informática, Universidad Veracruzana, Xalapa 91190, Veracruz, Mexico

**Keywords:** optical guidance, cells projection, actin

## Abstract

Highly focused near-infrared (NIR) lasers have been used to induce fibroblast and neuron protrusions in a technique called optical guidance. However, little is known about the biochemical and biophysical effects that the laser provokes in the cell and optimal protocols of stimulation have not yet been established. Using intermittent NIR laser radiation and multivariate time series representations of cell leading edge movement, we analyzed the direction and velocity of cell protrusions. We found that the orientation and advance of PC12 neuron phenotype cells and 3T3 fibroblasts protrusions remain after the laser is turned off, but the observed increase in velocity stops when radiation ceases. For an increase in the speed and distance of cell protrusions by NIR laser irradiation, the cell leading edge needs to be advancing prior to the stimulation, and NIR irradiation does not enable the cell to switch between retracting and advancing states. Using timelapse imaging of actin-GFP, we observed that NIR irradiation induces a faster recruitment of actin, promoting filament formation at the induced cell protrusions. These results provide fresh evidence to understand the phenomenon of the optical guidance of cell protrusions.

## 1. Introduction

Highly focused near-infrared lasers (NIR) have been reported to attract or repel cell protrusions in a technique that has been called optical guidance. This has been described for fibroblasts [1,2,3] and neurons [4,5,6,7,8,9,10,11,12] using several different laser configurations, wavelengths, and powers. Although different mechanisms have been suggested to take part in the cellular response, limited experimental evidence has been reported and no model has yet gained consensus. Some authors have suggested that the gradient of the NIR radiation induces cytoskeleton polymerization at the site of stimulation, enhancing actin filament formation and the regulation of filopodia and lamellipodia at the leading edge of the stimulated neurons [6,8]. An alternative hypothesis suggests that the absorption of NIR light can lead to a temperature rise. Theoretical calculations have shown that an 800 nm laser with a power of 100 mW would increase the temperature of water at the laser spot by 0.32 °C [13]. A similar increase was obtained for cells under NIR optical trapping [14]. Interestingly, an increase in the neurite outgrowth rate has been observed because of a temperature gradient created using a 1455 nm laser to heat the proximity of neuronal cells up to 10 °C [15]. Such a high temperature increase is reached because at that wavelength, the absorption coefficient of water is more than 100 times larger than at 976 nm or 810 nm [16], which are the wavelengths used in our study. In these micro heating experiments [15], an increase in intracellular Ca^2+^ was observed and the projection of growth cone was driven by a predominant induction of microtubules polymerization versus actin polymerization. Optical guidance is an interesting approach as a non-invasive method to guide cell projection; however, a more thorough study of the conditions and protocols of stimulation and a deeper comprehension of the cellular mechanisms involved are needed. The variability and complexity of neuronal growth cone dynamics make this goal a difficult one to achieve. During cell projection, plasma membrane protrusions are formed at the leading edge in the form of lamellipodia and filopodia. The former are rich in actin filaments organized as a branched filaments network, while the latter are longer protrusions with parallel actin filaments [17]. In both cases, complex interactions of actin-binding proteins, GTPase, and cell adhesion proteins [18] determine actin polymerization and depolymerization, which influence the elongation or retraction of the cell leading edge. Cell spreading is a stochastic process where a constant protrusion and retraction cycle is observed [18]. Retrograde actin flow, which is driven by actomyosin-mediated contractility, is fundamental for cell advance [19,20]. Understanding the cellular mechanisms that underlie optical guidance is a pending task that is scarcely being approached. In this paper, we analyze the optical guidance of PC12 neuron phenotype cells’ growth cones, and the effects of intermittent irradiation on the cell protrusion and velocity of cell edge projection. The measurements of cell edge velocities are analyzed with multivariate time series. Finally, cytoskeleton dynamics at the NIR-stimulated protrusion are studied using confocal images of actin-GFP.

## 2. Materials and Methods

### 2.1. Cell Culture

Pheochromocytoma PC12 cells were cultured on Dulbecco’s modified Eagle’s medium (DMEM) (Gibco, Thermo Fischer Scientific, Waltham, MA, USA) plus 5% (*v*/*v*) of fetal bovine serum (FBS) (Invitrogen, Thermo Fischer Scientific, Waltham, MA, USA) and inactivated 5% (*v*/*v*) horse serum (HS) (Invitrogen), plus 1% (*v*/*v*) of penicillin/streptomycin (100 units/mL and 100 μg/mL, respectively) (Invitrogen). For PC12 differentiation into a neural phenotype, cells were cultured on differentiation medium containing DMEM plus 1% of HS and 1% of antibiotics, plus 100 ng/mL of neural growth factor (NGF2S) (Sigma, St. Louis, MO, USA). For PC12 observations at the microscope, cells were cultured on collagen type I (2 µg/mL)-covered coverslips. Briefly, a solution of collagen type I (Advanced Biomatrix, San Diego CA, USA) was added to previously sterilized No. 1 coverslips, and was incubated for at least 1 h at 37 °C; the coverslips were washed once with phosphate-buffered saline (PBS) solution and maintained in PBS until their use. The cells were seeded in collagen-covered coverslips and maintained for 3–4 days on differentiation medium, until cells with a neuronal phenotype were observed.

The 3T3 NIH cell line (3T3) was cultivated in high-glucose DMEM (Gibco, Thermo Fischer Scientific, Waltham, MA, USA) supplemented with 10% (*v*/*v*) FBS and 1% (*v*/*v*) penicillin/streptomycin. The cells were split every 3–4 d or at confluence. Each cell line was maintained in a 37 °C and 5% CO_2_ incubator. 

For experiments with differential interferometry contrast (DIC) microscopy, 25 × 10^3^ cells mL^−1^ for 3T3 and 75 × 10^3^ cells mL^−1^ for PC12 were attached to 25 mm coverslips coated with collagen. For experiments with confocal microscopy, the 3T3 cells were cultured in 25 mm coverslips coated with collagen or alternatively in glass-bottom 35 mm dishes at 20 × 10^3^ cells mL^−1^ for 2 days, with the PC12 cells being seeded for 3–5 days. For microscopy observation, coverslips with cells were mounted in a chamber with a controlled temperature at 37 °C and 5% CO_2_ and maintained with DMEM supplemented with FBS for 3T3 cells or with HS for PC12 cells. Each medium was buffered with 10 mM of HEPES (Sigma, St. Louis, MO, USA).

### 2.2. Expression of Actin-GFP

CellLight reagent (Molecular Probes, Thermo Fischer Scientific, Waltham, MA, USA) for the expression of actin-GFP was used according to manufacturer’s recommendations. Thus, 50 particles per cell (ppc) for actin-GFP expression were added to previously 3T3 cell-seeded coverslips in 1 mL of DMEM supplemented with FBS. Afterwards, the cells were grown for 48 h in a humidified 5% CO_2_ incubator at 37 °C. Live-cell imaging was carried out in DMEM without phenol red, containing 10% (*v*/*v*) FBS and 1% (*v*/*v*) of antibiotics, buffered with HEPES 10 nM. Four cells with actin-GFP expression under NIR irradiation were registered.

### 2.3. Optical Setup

The optical stimulation experiments were carried out using two different setups. For DIC images, the projection of cells was stimulated with a continuous-wave 976 nm laser (Thorlabs BL976-SAG300, Newton, NJ, USA) of 0–300 mW power coupled to an inverted microscope (Olympus IX81, Tokyo, Japan). Images were acquired with an Electron Multiplying Charge Couple Device camera (Luca R, Andor, UK) with 1002 × 1004 square pixels, each having a side of 8 μm on chip. The objective lens was a PlanApo 60×/1.40 NA oil immersion (Olympus, Tokyo, Japan). On the image, each pixel corresponds to 133 nm. The laser position was controlled by a steering mirror that was made the conjugate of the objective entrance pupil. The power at the objective exit was 40 mW. The confocal fluorescence imaging was conducted on a Nikon Confocal C1-Si inverted microscope. A Ti:Sapphire (Ti:sap) laser, operating in CW mode at a 810 nm wavelength, was positioned using a pair of galvanometric mirrors. The laser beam entered the microscope through the rear port and using a short-pass (Semrock FF720-SDiO1-25x36, Rochester, NY, USA) dichroic mirror (see Ref. [21] for more details). The power of the NIR laser was controlled and monitored by measuring the power of the reflection of a polarizing beam splitter (BS) cube when rotating a half-wave plate. Transmission images can be acquired either using the transmission detector when using the confocal unit or using a CMOS camera (DCC1545M-Thorlabs) when illuminating with the standard halogen lamp. During the experiments, the samples were mounted inside a chamber with the temperature held at 37 °C using a Tokai Hit stage top incubator. The software NIS-Elements version 4.10.00 (Nikon Instruments Microscopes and Digital Imaging Systems, Tokyo, Japan) was used for image acquisition and processed with FIJI ImageJ software^®^ 2.0.0 (National Institutes of Health, Bethesda, MD, USA) (image size 512 × 512). The actin-GFP was excited at 488 nm and the emitted fluorescence was collected through a 515/30 nm bandpass emission filter. The scanned images were recorded at a rate of 15 frames per minute. The dwell time was 15.3 μs. The scanner zoom factors used were 4.274 or 5.129.

### 2.4. Laser Stimulations and Live Cell Imaging

The cells were stimulated with a laser spot whose full width at half maximum was 3 μm on the image plane. The superficial power density was 5.7 mW$/{\mu m}^{2},\;$ if absorption is neglected, which is a justified assumption [22]. The laser was intentionally slightly out of focus to prevent optical trapping phenomena. Its position was controlled using the additional set of Galvo mirrors at the rear input of the microscope. The center of the laser spot was located 5 µm away from the leading edge of the growth cone or the lamellipodium. The position of the laser was adjusted to a few angular degrees above or below the forward direction when the leading edge came close to the laser. The optical stimulation experiments consisted of a 20 min period without laser stimulation (PREV), followed by two cycles of 20 min with the laser (ON) and 20 min without the laser (OFF). This sequence was adopted for both cell lines, PC12 and 3T3. For fluorescence images of the cytoskeleton, samples were recorded for several sequences.

### 2.5. Image Processing and Data Analysis

All images were processed using the FIJI ImageJ software [23]. The DIC images were filtered by the unsharp masking and threshold B/N tools, and the cell edges were delineated with the wand tracing tool and by manual adjustment of the contours. Pseudo-coloring of the images was achieved by selecting a lookup table and overlay was carried out with the merge channels tool. For confocal images, a series of confocal optical sections of 0.2 µm were projected using the maximum intensity projection option of selected z-stacks where the GFP signal was clearly observed. The smooth tool followed by brightness and contrast adjustment were applied at the same values for all of the images and conditions.

For the analysis of the cell images, we first define a reference point, O, for each cell in the first image of an acquisition set. The contour of the cell projection is determined, and a straight line, A, is fitted by linear regression to the collection of points that delineate the contour, using the MATLAB (MATLAB, Mathworks Inc., Natick, MA, USA) function named polyfit. The reference point, O, is set inside the cell image, 5 µm away from the cell contour along A. Then, the image was divided into zones separated by two lines. The first line passes through O and the location of the laser spot at the beginning of the first stimulation. The second line passes through O and is perpendicular to the first line. These lines define four quadrants. The first quadrant (0–90°) corresponds to the area stimulated with the laser. For a fixed laser, the stimulated zone was divided into two regions of 45° each. The area of the cell projection was measured using Fiji, at the beginning and end of each period (ON or OFF). The zones corresponding to the axon region were excluded from the measurement area. The cell projection evolution was assessed by the difference of the projection areas at the end and the beginning of a stimulation cycle. To determine whether a difference in the median values of the two data sets was statistically significant, we used the non-parametric Kruskal–Wallis test. We considered that the differences in the median values were statistically significant when the *p*-value of the test was lower than 0.05.

To calculate the speed of the cell leading edge projection, a set of radial lines that cross on point O and whose orientation varies in steps of 10° was determined. Measurements were obtained every minute, during one hundred minutes, under different periods when the NIR laser was ON and OFF, respectively (*n* = 3600 measurements per cell, complete data base *n* = 14,400 measurements). The speed of the cell leading edge projection along each angle was obtained by computing the difference of the distance from O to the edge between two consecutive images, divided by the time lapse that separated the two images. The values obtained when changing status (ON–OFF) were obviously discarded. The data were plotted with a multivariate time series diagram as described previously [24], using a program developed in R language. The measurements corresponding to the axon region were excluded from the plots.

The average distance, speed, and velocity were calculated as follows. The distance travelled by the membrane edge along a given angle between frame *n* − 1 and *n* is defined as

(1) $$d_{n}=\left|p_{n}-p_{n-1}\right|$$

where $p_{n}$ is the position in microns from point O along the line corresponding to the specific angle. The average distance over M timesteps is given by

(2) $$d_{avg}=\frac{1}{M}\overset{M}{\underset{i=1}{\sum }}d_{i}.$$

The average speed is obtained from the mean instantaneous speed:

(3) $$u_{avg}=\frac{1}{M}\overset{M}{\underset{i=1}{\sum }}u_{i}=\frac{1}{M}\overset{M}{\underset{i=1}{\sum }}d_{i}/\Delta t,$$

where the time interval between frames, Δt, is equal to 1 min. The average velocity, i.e., the directional speed, is obtained by dividing the change between the final and initial absolute positions over the total elapsed time:

(4) $$v_{avg}=\frac{p_{f}-p_{0}}{T}.$$

To analyze the actin-GFP average intensity change rates, kymographs were obtained from the z projection time lapse recorded images using the FIJI Reslice command, using a selected rectangle of 10 × 2 µm from the cell toward the laser spot; an equivalent area was selected at the time lapse images recorded without laser stimulation. An average of pixel intensity for each 1 µm of the selected area was obtained for each minute of the kymograph, and the speed of fluorescent change was calculated by obtaining the slope of the curve for each time point. To compare the data, the rate of fluorescent change was plotted against the distance toward the laser spot, or an equivalent area in non-irradiated conditions.

## 3. Results

### 3.1. Intermittent NIR Laser Stimulation on PC12 Cells with a Moving Laser Spot

**Figure 1:**
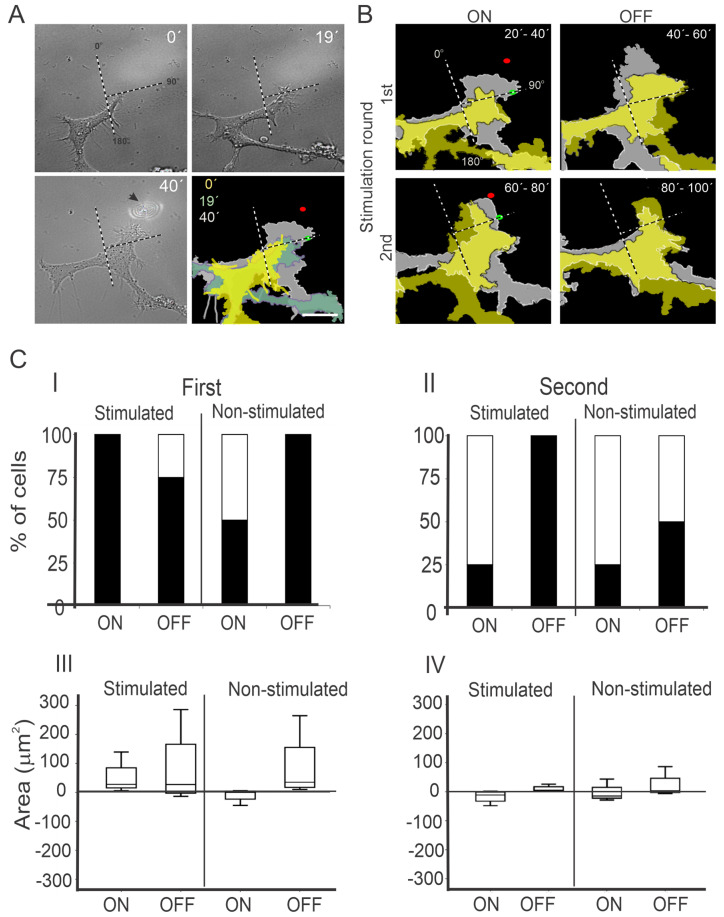
Optical guidance of PC12 cells during the ON and OFF period of laser stimulation. (**A**) Example of a time lapse of DIC microscopy images of PC12 cells obtained before NIR laser stimulation (T = 0′, T = 19′) and after 20 min of NIR laser irradiation (T = 40′). The arrow in the 40’ frame indicates the laser spot. The superposition of colored areas shows the direction of projection of the growth cones. (**B**) Example of growth cone projection while the laser was ON and OFF, respectively. The superimposed colored areas indicate the growth cone contour at beginning (yellow) and the end (gray) of the temporal periods indicated at the right-top corner of each frame. Dotted lines in (**A**) and (**B**) show the division in two zones (0–90° and 90–180°), green and red dots mark the position of the laser spot at the beginning and the end of the stimulation, respectively. (**C**) Quantification of PC12 growth cone projection. Subpanels I and II show the percentage of cells with increased (black) or decreased (white) growth cone area at stimulated (0–90°) and non-stimulated quadrants (90–180°), and during the laser ON and OFF periods at the first (I) and second (II) round of laser stimulation. III and IV show box-and-whisker diagrams of the difference of the growth cone areas at the end and beginning of the 20 min periods (refer to Section 2.4), at stimulated and non-stimulated quadrants, during the ON and OFF periods, in the first (III) and second (IV) round of laser stimulation. The scale bars in (**A**,**B**) represent 15 µm.

The PC12 cells were irradiated by a laser of 976 nm wavelength and 40 mW of power focused at the leading edge of the growth cone, during 20 min. The power was measured at the exit of the microscope objective. An example is illustrated in Figure 1A. The laser spot was displaced while the growth cone advanced, following an optical guidance irradiation scheme. We observed a sustained projection of the growth cone towards the laser spot, which was in the 0–90° quadrant. During the 20 min prior to the laser stimulation, the cell was growing towards a different direction, in the 90–180° quadrant.

To find out whether the direction of projection was maintained after the stimulation, the NIR laser was turned off for 20 min. We observed a long-lasting projection of the growth cone in the same quadrant during the OFF period (Figure 1B). When we turned on the laser for a second round of stimulation, the direction of projection was maintained; however, when the laser was off again, the cell edge projection stepped back, although no change in the direction of projection was evident (Figure 1B).

To quantify the changes in the growth cone areas, the difference of the areas from the first and last images was obtained for four different stimulated neurons.

#### 3.1.1. First NIR Irradiation

During the first irradiation, 100% of the cells increased their growth cone area on the stimulated quadrant (0–90°) versus 50% on the adjacent, non-stimulated quadrant (90–180°), as illustrated by the bars labeled ON in Figure 1C-I. The median values of the growth cone area difference between the end and the beginning of the stimulation were 27.68 and 0.312 µm^2^ on the stimulated and non-stimulated quadrants, respectively (Figure 1C-III, stimulated-ON, non-stimulated-ON), although no significant difference in the statistical sense was obtained (Kruskal–Wallis test, *p* > 0.05). When the laser was turned off, 75% of the cells increased their growth cone areas in the stimulated quadrant. The median values of the growth cone area difference in the stimulated quadrant between the end and the beginning of the 20 min periods of ON and OFF were 27.68 and 26.98 µm^2^, respectively (Figure 1C-III, stimulated ON and OFF), although the values were more largely dispersed when the laser was OFF.

#### 3.1.2. Second NIR Irradiation

During the second stimulation, only 25% of the cells increased their growth cone area during the ON period in both the stimulated and non-stimulated quadrants. However, in the OFF period, 100% and 50% of the cells increased their growth cone areas in the stimulated and non-stimulated regions, respectively (Figure 1C-II). The median values of the growth cone area variations are the same between the stimulated and non-stimulated zones. Interestingly, during the second round of stimulation in the stimulated quadrant, a negative median value was obtained at the ON period, whereas in the OFF period, the median area variation was positive (−11.45 vs. 5.32 µm^2^), suggesting that a delayed effect of the laser could be present until the OFF period (Figure 1C-IV stimulated ON and OFF).

### 3.2. Intermittent NIR Laser Stimulation on PC12 Cells with a Static Laser Spot

**Figure 2:**
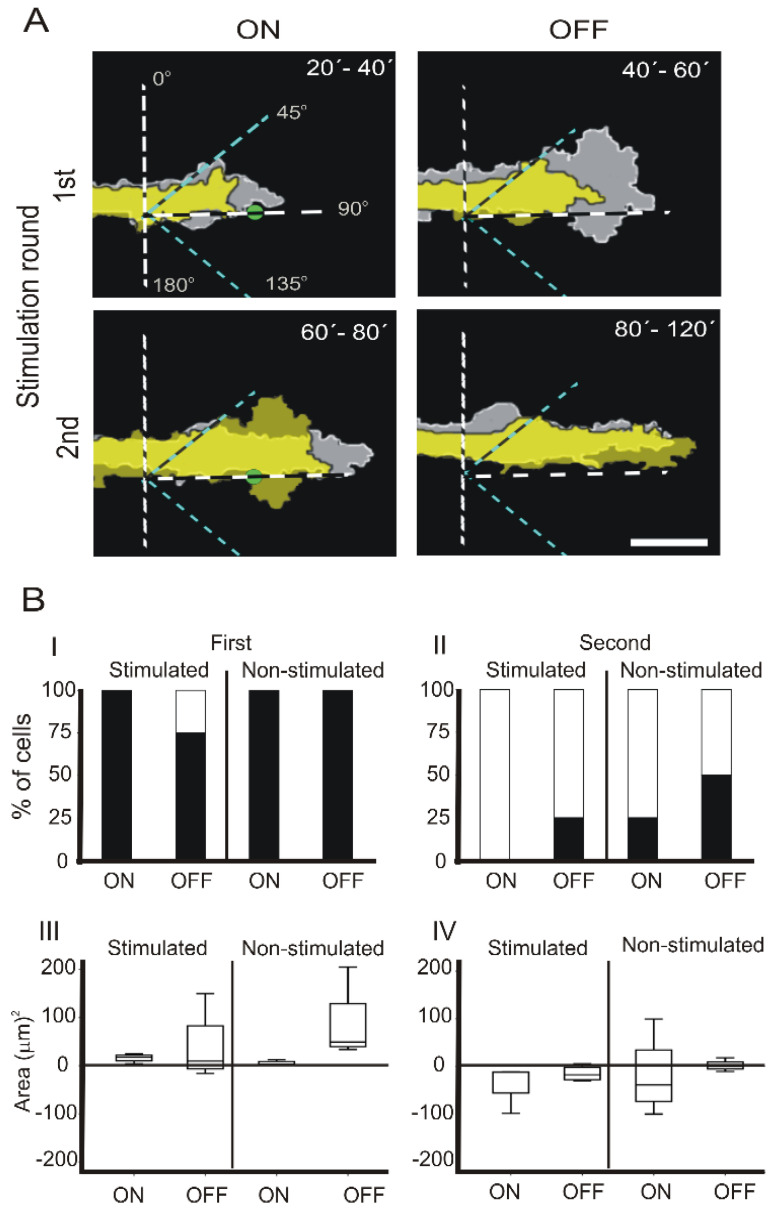
PC12 growth cone projections with fixed laser position at the on and off period of laser stimulation. (**A**) Similar to Figure 1B, with static laser. Stimulated quadrants, 45–135°, delimited with blue dotted lines, and non-stimulated zones. Green dots mark the position of the laser during all the stimulation period. (**B**) Similar to Figure 1C. The scale bar in A represents 15 µm.

In order to study the effects of NIR stimulation at a fixed position in the growth cone, and investigate whether or not the fixed NIR laser could sustain and/or increase cell projection once the laser was turned off, the growth cones were irradiated without moving the laser for 20 min followed by 20 min without irradiation. The differences in the projected areas were measured and compared between ON and OFF periods at the stimulated and non-stimulated quadrants (Figure 2). We observed that the growth cone edge surpassed the NIR laser location during the first 20 min of irradiation, and once the laser was off, the growth cone edge continued to advance (Figure 2A, 1st).

In the second round of laser stimulation, the growth cone continued to advance in some cells, although the total area decreased due to the narrowing to the cell at the back of the growth cone once the laser was off (Figure 2A, 2nd). When we compared the percentage and the median of the differences of the projected areas, we found the following results.

#### 3.2.1. First NIR Irradiation

We observed that 100% of the stimulated cells increased their area while the laser was irradiating and 75% of the cells maintained the increase once the laser was OFF, in a similar proportion to those in the experiments with the displaced laser (Figure 2B, I). In addition, 100% of the cells increased their area in the regions adjacent to the irradiated quadrant during the ON and OFF periods (Figure 2B, I).

#### 3.2.2. Second NIR Irradiation

During the second stimulation, no increase in the projected areas was observed in the ON period and only 25% of the cells increased the area once the laser was off (Figure 2B, II). Like in the guidance scheme, no significant differences were obtained when the medians were compared due to the high data dispersion (Figure 2B, III and IV).

### 3.3. Laser Stimulation Can Increase the Velocity of Cell Projection

To analyze the projection of growth cones with and without laser stimulation in more detail, the velocity of projection along the directions separated by 10° each was calculated by measuring the distance traveled by the cell membrane projection along those directions, during the one-minute period that separates each video frame. To visualize the data, we used a multivariate time series plot where the velocities were assigned a color code among three categories: green, purple, and gray, corresponding to positive, negative, and null velocities, respectively.

**Figure 3:**
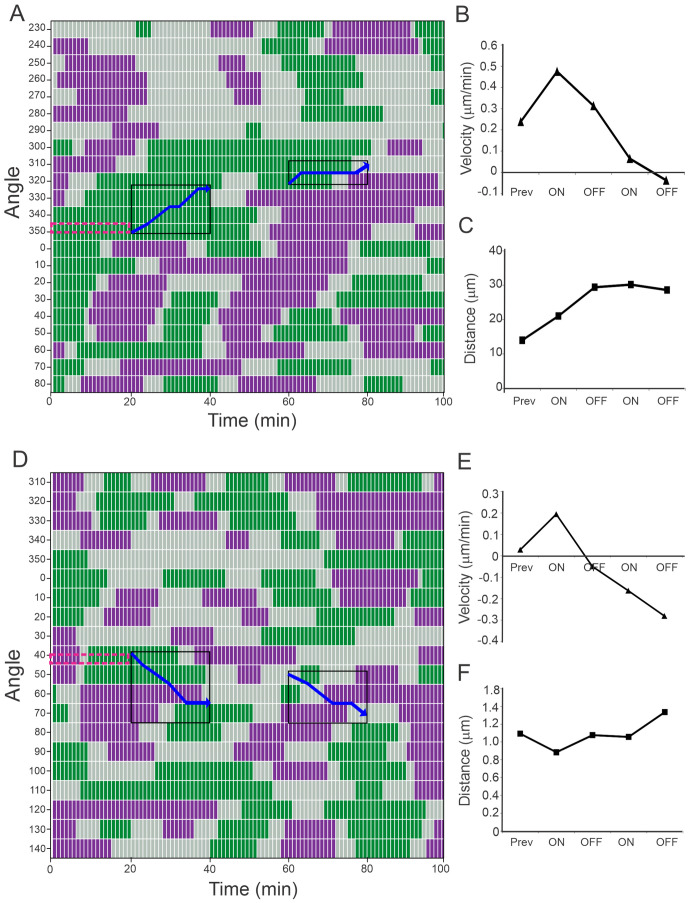
Quantification of PC12 growth cone velocity of projection. Multivariate time series plot of discretized velocities of projection at different angles. (**A**–**C**, cell #1) and (**D**–**F**, cell #2). Green, purple, and gray correspond to positive, negative, and null velocities, respectively. Black squares indicate the angles where the laser was located and blue arrows the displacement of the spot. In both cells, positive velocities were registered for at least 10 min previous to the first round of irradiation, at the angles of the spot location (red dotted squares). (**B**,**E**) show the median values of the velocities of projection, and (**C**,**F**) the median values of the cumulative distance, in both cases at the angles where the spots were located during the irradiated (ON), non-irradiated (OFF), or previous to irradiation (PREV) periods.

**Figure 4:**
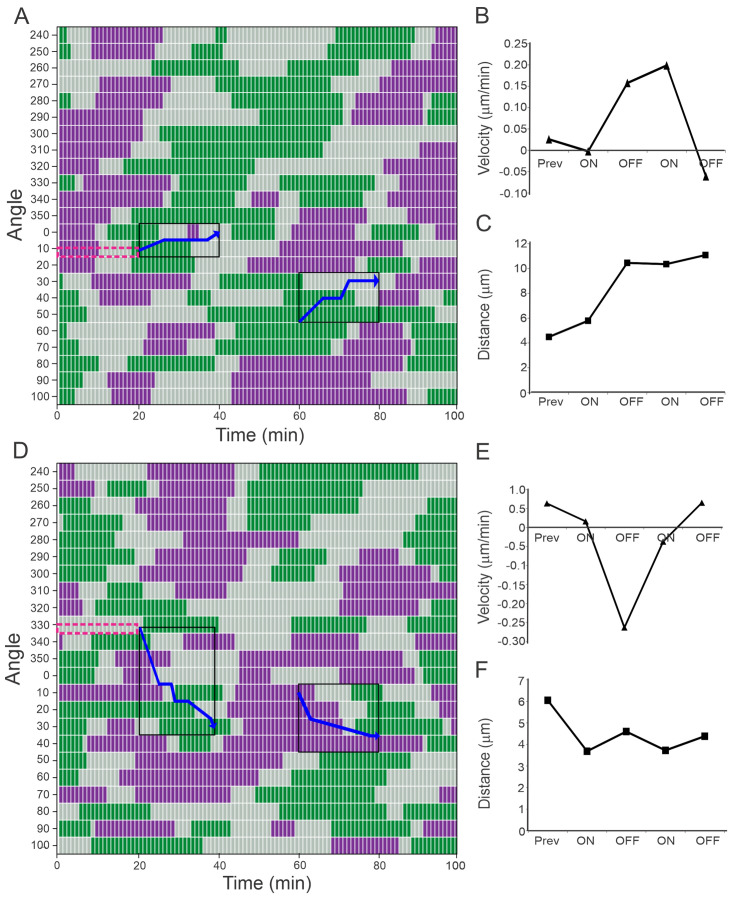
Similar to Figure 3, for cells #3 and #4 (**A**–**C**) and (**D**–**F**), respectively.

This representation evidences that cells are constantly alternating between advance and retraction movements, as previously reported [18]. In the color code charts of Figure 3 and Figure 4, each row corresponds to one angle and each column corresponds to one time. For example, in Figure 3A, one can see that along the line at 280 degrees, the cell membrane started with a retraction (purple), and then at *t* = 20 min, the membrane stood still (gray) and at *t* = 60 min the membrane started to protrude (green) for an approximately 20 min period.

The laser provoked an increase in the mean velocity of cell projection at the irradiated angles, depending on the cell status previous to the irradiation. Cells that had a green region before the irradiation angles (Figure 3A,D, red squares) increased their mean velocity of projection in the first ON period (Figure 3B,E), while cells that had a gray region or just began to advance previous to the laser irradiation (Figure 4A,D, red squares) did not increase and even decreased their mean velocity of projection once the laser was on.

When the laser was off after the first irradiation period that resulted in a velocity boost (Figure 3), a drop in mean velocity was observed; nevertheless, the cells continued their advancement, as shown in the distance plots, even when no increase in speed was observed again in the second round of stimulation (Figure 3B,C,E,F). In the case of the cells where no increase in velocity was observed during the first stimulation (Figure 4), the second stimulation provoked an increase in the projection velocity, even if that only means to decelerate the retraction (Figure 4B,E). The different behavior of the cells in Figure 3 and Figure 4 is probably a consequence of the different cell status before laser stimulation; while the cell membranes of Figure 3 were projecting with positive velocities before the NIR laser was turned on, those of Figure 4 were just beginning to project, or were static (see red squares in Figure 3A,D and Figure 4A,D).

Similar experiments were performed on 3T3 cells. We observed a clear acceleration of the cell projection as a result of the IR laser in both the first and the second stimulations (see Supplementary Figure S1).

**Figure 5:**
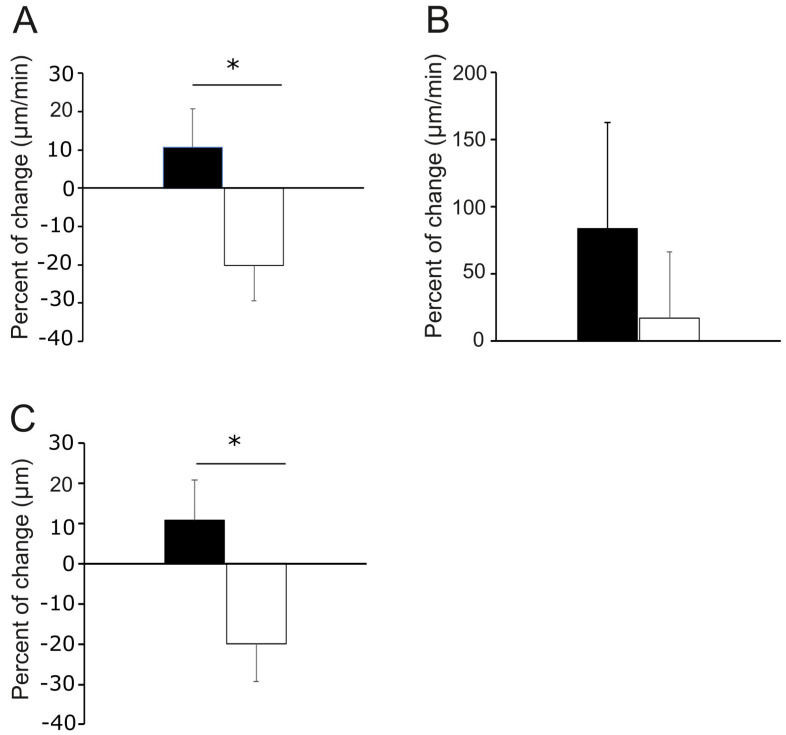
Percentage of change in membrane projection of PC12 active or static cells under laser irradiation. The percentage of change of (**A**) average speed, (**B**) average velocity, and (**C**) average distance was obtained from the irradiated regions of cell membranes before and after laser stimulation. Black columns correspond to cells that were projecting for at least five minutes before the beginning of laser irradiation (active); white columns correspond to cells that were immobile or retracting at the beginning of the laser irradiation (static). Bars represent the standard error. Asterisk (*) indicates statistically significant difference between the two conditions (Student’s *t* test *p* < 0.05). *n* = 8 cells per condition.

To verify whether the cell status before laser stimulation modifies the response to laser irradiation, we analyzed the percentage of change in the average distance, speed, and velocity (defined by Equations (2)–(4)) of the irradiated cell regions during the first irradiation in 16 cells. Eight of them were projecting their membranes at least five minutes before stimulation (active status), and eight were either immobile or retracting (static status). The results show a positive percentage of change in all of the parameters in active cells and a negative percent of change for static cells in speed and distance (Figure 5A–C). The comparison between the active and static cells was only significant for speed and distance (Figure 5A,C). The results suggest that the laser has a different effect on static and active cells. On static cells, the laser reduces, on average, the movement of the cell membrane, whereas on active cells, the laser enhances it. Moreover, the laser tends to increase the overall projection distance of cell membranes in active irradiated cells, and it promotes the retraction of cell membranes in static cells.

### 3.4. Effects of Laser Stimulation on Actin Cytoskeleton

**Figure 6:**
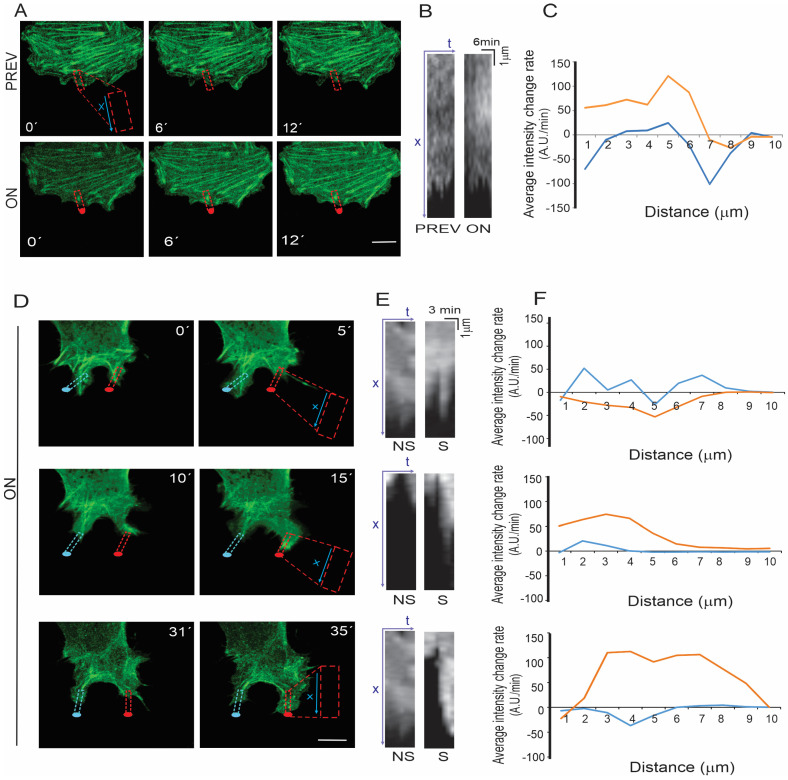
Effects of laser stimulation on actin-GFP. (**A**) Selected images of a 3T3 cell expressing actin-GFP during 12 min previous to its stimulation (PREV) and while laser-stimulated (ON). Red dots indicate the position of the laser spot during the ON period. Dotted lines indicate the rectangular area selected for the kymograph analysis. Widened copies of the red rectangles are shown to indicate the axis of the position dimension (X) of the kymographs. (**B**) Kymographs obtained from the cell area adjacent to the laser spot (ON) or from an equivalent area during the period of time without the laser stimulation (PREV). (**C**) Temporal fluorescence intensity change in the kymographs averaged over the observation period (0–12 min), as a function of the position along the x axis along the region of interest shown by the dashed rectangles. The non-stimulated condition is represented by a blue line, while the orange line corresponds to the laser-stimulated condition. (**D**) Selected images of a 3T3 cell expressing actin-GFP under laser stimulation (ON). Laser spot (red dots) was displaced, while the cell advanced at different time intervals. Red dotted lines indicate the selected areas for the kymograph computations at the time periods when the cell was projecting (0–5, 10–15, and 31–35 min). Widened copies of the red rectangles are shown to indicate the axis of the position dimension (X) of the kymographs. Blue dotted lines indicate an equivalent area in the same cell without laser stimulation. (**E**) Kymographs obtained from non-stimulated (NS) or laser stimulated areas (S). (**F**) Similar to **C**, for the kymographs shown in (**E**). Scale bars in (**A**) and (**D**) correspond to 10 µm.

The cytoskeleton has been scarcely studied in cells stimulated to project by a NIR laser. We used 3T3 cells to study the actin dynamics due to the larger size of their lamellipodia and filopodia. By comparing changes in the average fluorescence signal in a rectangular area of 10 × 2 µm^2^ of a cell protrusion, before and during laser stimulation (Figure 6A,B, PREV and ON), we observed a faster rate of change of the actin-GFP signal during 12 min of laser stimulation than the same period of time and area before NIR stimulation (Figure 6C). In both cases, the rate of intensity change decreases at the cell leading edge. At the NIR laser stimulated protrusion, a filament-shaped actin accumulation that grows towards the cell leading edge and perpendicular to the stress fibers is observed; in the same area, before laser stimulation, we observed actin-GFP signal fluctuation among diffuse staining and thin actin filaments (Figure 6A).

In the actin-GFP images obtained during optical guidance and laser spot displacement, cell projections alternate between retraction and advance movements, as described before. Figure 6D–F evidence that when the NIR laser stimulates a protrusion, the actin-GFP signal increases faster than without the laser stimulation, but only after 5 min of irradiation. The fluorescent signal at the irradiated protrusions corresponds to filament-like actin-GFP accumulation.

## 4. Discussion and Conclusions

Our results show that under NIR laser irradiation, actin-GFP was recruited faster in a filament organized shape, which invades the protrusion cell leading edge, suggesting that the laser-stimulated protrusions could be driven by accelerated actin recruitment and faster filament formation. The presence of actin filament bundles at the NIR laser-stimulated filopodia of growth cones has previously been reported; however, these were fixed cells, and no actin dynamics were observed [6]. It has been well characterized in fibroblasts and neurons that actin polymerization at the leading edge couples to adhesion complexes, counteracting retrograde actin flows and turning into traction forces to pull the protrusions forward [18,20,25,26]. The observed recruitment of actin at the cell protrusion of irradiated cells might be improving actin coupling at the leading edge, and therefore not only directing the protrusion toward the laser, but accelerating its projection.

On the other hand, the plots of the projection velocities confirm that the leading edge of the growth cones has a stochastic behavior of protrusion and retraction, as has been extensively characterized for several cell types [18,27,28,29]. However, the laser irradiation does not seem to induce a change of retraction or protrusion states; rather, it induces an increase in the mean velocity of the irradiated zone, but only when the cell was already advancing prior to the NIR irradiation. Betz et al. (2007) reported a similar stochastic behavior of NG108 neuronal cells and observed that irradiation with an 800 nm laser did not change protrusion–retraction phases, but increased the edge velocity on stationary growth cones, while in advancing ones, protrusion was favored without a velocity increase [30].

Stochastic cell edge behavior is linked to the polymerization–depolymerization cycles of actin filaments. A decrease in the advance–retraction events rate is observed when cells are induced to project [18]. Cell leading edge projection is enhanced when actin rearward flow and cell contractility increase, leading to the reinforcement of cell adhesion [18,31,32]. The velocity of projection is influenced by an increase in actin retrograde flow in the cell with a polarized lamellipodium or protrusion. When the polarized conformation is abolished, no increase in speed was observed [32]. Interestingly, our results indicate that a previous advancing state is necessary for an increase in velocity to occur under the NIR stimulation, which could be linked to a previous state that favors polarized protrusions. One of the relevant findings in this work is, therefore, that the effect of the laser depends on the initial state of the cell projection. It is interesting that the stimulation of neurite projection by red light irradiation is enhanced when myosin II, a protein involved in growth cone contraction, is inhibited, suggesting that photo-biomodulation effects depend on the contractility state of growth cones [33]. A non-homogeneous or “synchronized” cell state at the moment of the stimulation by NIR could explain a high data dispersion, which has also been reported even when the number of cells is bigger than that reported in here [26]. We suggest that future experiments use “synchronized” cell populations in order to make a confident statistical analysis. 

On the other hand, our research explores the effects of re-irradiation with a laser for the first time, observing that the laser effect on the direction of projection and the advance of the leading edge can be maintained after the laser irradiation stops. However, the velocity of projection decreases once the laser is off. Moreover, once the laser is turned on again, no recovery of the velocity is observed, suggesting that the laser effect is limited in time or refractory for longer periods of time. Interestingly, in cells where no laser effects are observed during the first round of stimulation, a delayed effect is observed after the laser is off or until the second round of laser stimulation is applied, suggesting that the first irradiation round could influence cells’ molecular conformation and/or cell polarity as discussed before, and therefore the effect is held once the cell state is somehow ready to respond. A more profound characterization of the two states of the cells, regarding their protrusion, polarization, actin, and adhesion conformation, will be necessary to obtain a deeper insight into the molecular mechanisms that determine the NIR laser effects on cell protrusion.

It is worth mentioning that NIR monochromatic light is also used in optogenetics, which employs natural and engineered photoreceptors, mostly of microbial origin, to be genetically introduced into the cells of interest, which then become sensitive to light [34]. For example, receptor tyrosine kinases, which play an important role in a variety of cellular processes including growth, motility, differentiation, and metabolism, can be regulated with NIR light [35]. An NIR laser has also been used for the rapid thermogenetic control of neuronal activity in fruit Drosophila [36]. An interesting review of the methods for controlling electrical activity in nerve cells is give in [37,38].

In conclusion, our results show that the induction of cell protrusion using a focused NIR laser depends on the previous cell projection status, and the increase in the velocity of projection may be coupled to accelerate actin filament polymerization oriented parallel to the cell protrusion. The direction and advance of cell protrusion are maintained when the laser is off, but the velocity of projection is not. Even though this report contributes to the knowledge of the cellular mechanisms behind cell protrusion induced by an NIR laser, more detailed comprehension and protocols are needed to bring optical guidance towards a feasible option as a non-invasive technique for cell guidance in in vivo models.

## Data Availability

The data that support the findings of this study are available from the corresponding author upon reasonable request.

## Supplementary Materials

The following supporting information can be downloaded at: https://www.mdpi.com/article/10.3390/cells12040540/s1, Figure S1: Quantification of a 3T3 cell leading edge velocity of projection.
