# Supplementary material for: Infrared Laser Effects on Cell Projection Depend on Irradiation Intermittence and Cell Activity
Source: Cells. 2023 Feb 8;12(4):540. doi: 10.3390/cells12040540 (PMC9954793; doi:10.3390/cells12040540)
Supplement: Supplementary file 1 [file cells-12-00540-s001.zip › cells-2098330-supplementary.pdf]

Supplementary material

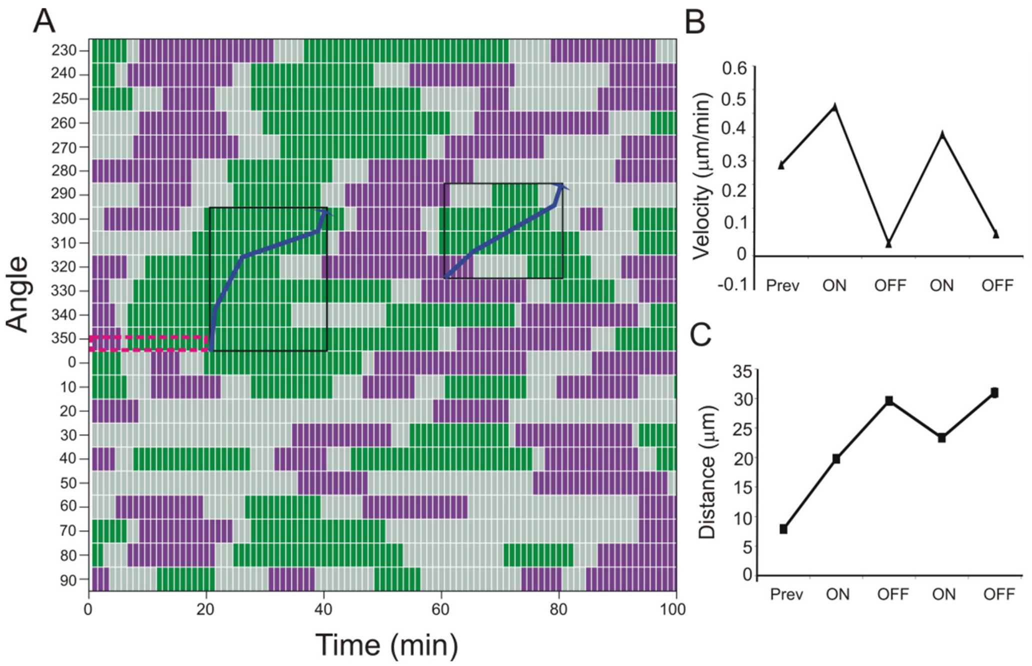

Supplementary Figure S1. Quantification of a 3T3 cell leading edge velocity of projection. Multivariate time series plot of discretised velocities of projection at different angles (A), Green, purple and grey correspond to positive, negative and null velocities, respectively. Black squares indicate the angles where the laser was located and blue arrows the displacement of the spot. Positive velocities were registered for at least 10 minutes previous to the first round of irradiation at the angles of the spot location (red dotted squares). (B) shows the median projection velocities, and (C) shows the median of the cumulative distance at the angles where the spot were located, during the irradiated (ON), non-irradiated (OFF), or previous to irradiation (PREV) periods.
